# Enhancing prehospital analgesia: addressing methodological concerns and proposing the START-A mnemonic

**DOI:** 10.1186/s13049-024-01220-2

**Published:** 2024-06-06

**Authors:** Rohat AK, Ali Cankut Tatliparmak, Sarper Yilmaz

**Affiliations:** 1 Dept. of Emergency Medicine, University of Health Sciences, Kartal Dr. Lutfi Kirdar City Hospital, Istanbul, Turkey; 2 Dept. of Emergency Medicine, Uskudar University Faculty of Medicine, Istanbul, Turkey; 3 Dept. of Emergency Medicine, University of Health Sciences, Kartal Dr. Lutfi Kirdar City Hospital, Istanbul, Turkey

Dear Editor,

Deslandes and colleagues’ article, “Effectiveness and safety of prehospital analgesia with nalbuphine and paracetamol versus morphine in by paramedics - an observational study,” addresses significant clinical practice issues [1]. The study compares the analgesic efficacy and safety of nalbuphine + paracetamol and morphine. The data presented offer valuable insights for improving analgesia management in prehospital settings, yet they come with some methodological limitations.

The observational and retrospective design of the study might introduce biases in treatment assignments and necessitates cautious interpretation of the results. The substantial difference in patient numbers between the nalbuphine + paracetamol group and the morphine group (1,635 vs. 173) could impact the reliability of statistical analyses. Additionally, conducting the study in different regions and over different time periods might create further biases.

The study also addresses the issue of oligoanalgesia in prehospital analgesia practices. Oligoanalgesia, frequently encountered in emergency services, arises when patients do not receive adequate pain management. Overcoming this requires regular training for healthcare personnel in pain assessment and management, the development of pain management protocols, and the encouragement of multidisciplinary approaches [2].

At this point, we would like to suggest the “START-A” mnemonic that we developed for use in field triage for analgesia [3]. START-A offers simple and easy-to-remember steps to ensure rapid and effective analgesic treatment. This mnemonic could standardize pain management and help prevent oligoanalgesia. START, originally a triage system used in disaster situations (Simple Triage and Rapid Treatment), adapted as START-A (A stands for analgesia) in prehospital settings, especially in emergency and disaster scenarios, could enhance the mnemonic’s recognizability and motivate healthcare professionals to employ this method more broadly.

Comparing the pharmacokinetic properties of nalbuphine and morphine, nalbuphine typically begins to take effect within 2–3 min, whereas morphine takes about 5 min to start working. Both drugs have similar durations of effect; however, due to nalbuphine’s lower risk of respiratory depression, it is preferable for patients with respiratory issues. Additionally, nalbuphine’s ceiling effect reduces the risk of overdose and enhances safety in prehospital use [4, 5].

In terms of analgesic efficacy, the nalbuphine + paracetamol group achieved lower pain scores and a higher likelihood of reaching Numeric-Rating-Scale (NRS) < 4 at hospital handover compared to the morphine group. However, considering the pharmacokinetic profiles of the analgesics, nalbuphine’s rapid onset may be more suitable for prehospital environments and could influence the outcomes.

Regarding complications, although nalbuphine + paracetamol was associated with fewer complications, the retrospective nature of the study might have missed documenting mild or quickly resolving complications, which should be considered when interpreting these results.

In conclusion, Deslandes and colleagues’ study provides significant findings in the field of prehospital analgesia. However, the limitations arising from the study’s design should be considered, and the results should be interpreted in this context. Future research validating these findings through randomized controlled designs will contribute to the advancement of the field.

## Data Availability

Not applicable.

## Declarations

### Meetings

The article has not been presented in any organization.
